# Supplementary material for: Prognostic modeling in head and neck cancer: deep learning or handcrafted radiomics?
Source: BJR Artif Intell. 2025 Jul 15;2(1):ubaf008. doi: 10.1093/bjrai/ubaf008 (PMC13045703; doi:10.1093/bjrai/ubaf008)
Supplement: ubaf008_Supplementary_Data [file ubaf008_supplementary_data.zip › Supplementary-2.docx]

| **TRIPOD + AI** | | **Review -**  **Assessment criteria** | **Reasons for non-inclusion in Review -**  **Assessment criteria** |
| --- | --- | --- | --- |
| **Introduction** | *Background* | Not Included | Included as part of study selection |
|  | *Objectives* | Not Included | Included as part of study selection |
| **Methods** | *Data* | Not Included | Included as part of study selection, refer Table 1 |
|  | *Participants* | Not Included | Included as part of study selection,  refer Table 1 |
|  | *Data preparation* | 3 |  |
|  | *Outcome* | Not Included | Included as part of study selection,  refer Table 2 |
|  | *Predictors* | 4 |  |
|  | *Sample size* | Not Included | Included as part of study selection,  refer Table 2 |
|  | *Missing data* | 2 |  |
|  | *Analytical methods* | 3, 4 |  |
|  | *Class imbalance* | 2 |  |
|  | *Fairness* | Not Included |  |
|  | *Model output* | 6 |  |
|  | *Training versus*  *Evaluation* | Not Included | Included as part of study selection (Tripod 3 or 4), refer Table 1 |
|  | *Ethical approval* | NA |  |
| **Open Science** | *Funding* | NA |  |
|  | *Conflicts of*  *interest* | NA |  |
|  | *Protocol* | 1 |  |
|  | *Registration* | 1 |  |
|  | *Data sharing* | Not Included | Included as part of study selection,  refer Table 1 |
|  | *Code sharing* | Not Included | Included as part of study selection,  refer Table 1 |
| **Patient & Public Involvement** | *Patient & Public*  *Involvement* | NA |  |
| **Results** | *Participants* | Not Included | Included as part of study selection,  refer Table 1 |
|  | *Model development* | 4 |  |
|  | *Model*  *specification* | Not Included | Included as part of study selection,  refer Table 2 |
|  | *Model*  *performance* | 4 |  |
|  | *Model updating* | 4 |  |
| **Discussion** | *Interpretation* | 5 |  |
|  | *Limitations* | NA |  |
|  | *Usability of the*  *model in the*  *context of current*  *care* | 8 |  |

| **ESR Radiomics guidelines** | | **Review -**  **Assessment criteria** | **Reason** |
| --- | --- | --- | --- |
| **Features** | *Image pre-processing* | 3 |  |
|  | *Feature extraction parameters* | 2 |  |
|  | *Image filters for higher-order radiomic features* | 3 |  |
|  | *Intuitions for features interpretability* | 5 |  |
|  | *Univariable analysis* | 4 |  |
| **Model development** | *Data partitioning* | Not included | Included as part of study selection (Tripod 3 or 4), refer Table 1 |
|  | *Outcome parameter selection* | Not included | Included as part of study selection (Tripod 3 or 4), refer Table 2 |
|  | *Model comparison* | 8 |  |
|  | *Model fine-tuning and assessment* | 4 |  |
|  | *Calibration of models/classifier* | Not Included |  |
|  | *Model explainability* | 5 |  |
